# Association analysis of retinoic acid receptor beta (*RARβ*) gene with high myopia in Chinese subjects

**Published:** 2010-05-13

**Authors:** Yang Ding, Xiaoyan Chen, Dongsheng Yan, Anquan Xue, Fan Lu, Jia Qu, Xiangtian Zhou

**Affiliations:** 1 School of Optometry and Ophthalmology and Eye Hospital, Wenzhou Medical College. Wenzhou, Zhejiang, China; 2 State Key Laboratory Cultivation Base and Key Laboratory of Vision Science, Ministry of Health P.R.China and Zhejiang Provincial Key Laboratory of Ophthalmology and Optometry. Wenzhou, Zhejiang, China

## Abstract

**Purpose:**

High myopia or pathological myopia is a common refractive error. Individuals with high myopia are subject to increased risk of serious eye complications. Accumulating evidence has demonstrated the role for heritability in ocular growth and in the development of high myopia. Retinoic acid and retinoic acid receptors play important roles in ocular development and in experimentally induced myopia. The purpose of this study was to determine if high myopia is associated with single nucleotide polymorphism (SNP) variants in the retinoic acid receptor beta (*RARβ*) gene in Chinese subjects.

**Methods:**

DNA samples were purified from venous lymphocytes of 175 unrelated Chinese patients with high myopia (less than −8.00 diopters) and 101 Chinese control subjects without high myopia (±1.00 diopters). Direct nucleotide sequence analysis in the RARβ gene was performed, and the detected variations were further confirmed by reverse sequencing. Allelic frequencies of all detected SNPs were assessed for Hardy–Weinberg equilibrium.

**Results:**

Five variations in *RARβ* were detected in Chinese subjects with high myopia, including 32574G>A, 32629G>A, 32645C>T, 32647T>G, and 151973C>T, of which only 32647T>G (NCBI notes as rs58244688 and rs2067964) had already been reported. The majority of SNP genotypes were heterozygous. While 32647T>G, 32629G>A, and 32645C>T were located in introns and 32574G>A and 151973C>T were located in coding regions, none of the SNPs affected the amino acid sequence. In the present study, no evidence of association was found between variations in the nucleotide sequence of *RARβ* and high myopia.

**Conclusions:**

Five SNP variants in *RARβ* were detected in Chinese subjects with high myopia, none of them were associated significantly with high myopia. Further studies are needed to identify which genes are responsible for high myopia.

## Introduction

High myopia is a major cause of visual impairment worldwide, especially in certain young Asian populations such as Chinese, Japanese, and Singaporean [1]. The development of high myopia is associated with excessive axial elongation, scleral thinning, and enlargement of the vitreous chamber depth, all of which are assumed to be the result of scleral remodeling [2,3]. People with high myopia are more likely to have other pathologic changes including primary open angle glaucoma, retinal detachment, macular degeneration, and subretinal hemorrhage [4,5]. High myopia is a complex eye disease involving genetic and environmental factors. While the exact mechanism underlying this abnormal ocular development is still unclear, there is genomic and clinical evidence in different ethnic populations that genetics plays a role in its development [6-10]. Though several mutation genes have been mapped in autosomal dominant or X-recessive inherited high myopia, no specific gene has yet identified [11-17].

All-trans retinoic acid (RA), dopamine, fibroblast growth factor, and transforming growth factor beta are implicated in the control of scleral remodeling and the development of myopia [18-22]. Different from the other candidate etiologies, changes in RA concentrations show sign of defocus selectivity, consistent with the morphological changes of the eye [18]. After visual deprivation in the chick, expression of RA increases in the retina, and the retinoic acid receptor beta (RARβ) is upregulated in the sclera [18,20,23]. Feeding RA to guinea pigs and chicks causes a rapid increase in ocular elongation [24,25]. RA also decreases scleral glycosaminoglycan synthesis while increasing vitreous chamber length during monocular deprivation in marmosets [26]. RA can elicit certain biologic responses by binding to and activating specific retinoic acid receptors (RARs) and the retinoid X receptors (RXRs) [27,28]. In our previous study, we found that six retinoic acid receptor subtypes (RARα, RARβ, RARγ, RXRα, RXRβ, and RXRγ) are present in cultured human scleral fibroblasts, and the proliferation of scleral fibroblasts is inhibited by retinoic acid [29]. We hypothesized that RARs are good candidate genes for myopia, especially high myopia. However a recent study found no association between *RARα* and either myopia or hypermetropia [30]. Therefore, the purpose of this study was to determine if there are significant associations between high myopia and single nucleotide polymorphism (SNP) variants in the *RARβ* exons of Chinese subjects.

## Methods

### Subjects

A total of 175 unrelated Chinese subjects with high myopia, having a spherical power of less than −8.00 diopters (D) in both eyes, were recruited for this study. The age range of the high myopia subjects examined was 7–68 years, and the average age was 34.2 years (Table 1). The average spherical power and the average cylindrical power were mentioned in Table 1. The subjects were from the Optometry Eye Hospital of Wenzhou Medical College, Wenzhou, China and were matched with 101 ethnically and socially similar, unrelated control subjects with refractive errors within ±1.00 D in each eye. None of the subjects had any known ocular, genetic, or systemic connective tissue disorders associated with myopia. The study protocol had the approval of the Human Subjects Ethics Committees of Wenzhou Medical College and Eye Hospital, Wenzhou, China. This study also adhered to the tenets of the Declaration of Helsinki with subsequent revisions. Written consent was obtained from every subject after being fully informed of the purpose and procedures of the study. Every participant received a complete ocular examination including visual acuity (Topcon RM-8800; Topcon Corp., Tokyo, Japan), slit-lamp evaluation of the anterior segment (Topcon SL-1E Slit Lamp; Topcon Corp.), dilated fundus examination (Heine Omega 180 Binocular Indirect Ophthalmoscope; Heine Optotechnik, Herrsching, Germany) and axial-length measurements (Zeiss IOL Master; Carl Zeiss Meditec, Jena, Germany).

**Table 1:** Ocular biometry data of the high myopic group.

** **	**High myopia group (n=175)**	
**Characteristics**	**Right eye**	**Left eye**
Spherical power (diopter)	-15.44±6.06	-15.18±6.28
Astigmatism (dopter)	-1.60±1.21	-1.85±1.26
Axial length (mm)	29.58±2.64	29.52±2.86
Age at exam (year)	34.2±14.2	

Values are mean ±standard deviation.

### DNA extraction and amplification

Genomic DNA for polymerase chain reaction (PCR) was extracted from 2 to 5 ml of peripheral venous blood from all participants. DNA was purified from lymphocytes according to the manufacturer’s instructions using a kit (BBI, Toronto, ON, Canada). Briefly, blood cells were lysed using lysis buffer and digested with proteinase K at 55 °C for 10 min. Then ethanol was added into the mixture and transferred into a spin column.  The flow through was discarded after centrifugation. Genomic DNA was eluted from the column. The purity of genomic DNA was determined by the measurement of absorbance ratio A260/280. The accession number for the *RARβ* sequence used for the construction of our primers was NT_022517. PCR, using primers designed to amplify *RARβ* (Table 2), was performed in a 50 μl volume with 31.25 μl double distilled H_2_O, 5 μl 10× PCR buffer, 3.5 μl 25 mmol/l MgCl_2_, 4 μl 2.5 mmol/l dNTP, 2 μl template DNA, 2 μl primer (Invitrogen, Shanghai, China), and 0.25 μl Ex Taq polymerase (Takara BIO Inc., Tokyo, Japan) following the manufacturer's recommendations (Perkin-Elmer, Norwalk, CT). Briefly, the PCR reaction was initiated at 94 °C for 5 min, followed by 35 cycles (94 °C for 15 s, 55 °C for 30 s, and 72 °C for 45 s) and ended at 72 °C for 10 min.

**Table 2:** Primers used for the amplification and sequencing of *RARβ*.

**Fragment**	**Direction**	**Primer sequence**	**PCR product (bp)**	**Exon**
RARβ 1	S	5′-GTGTGACAGAAGTAGTAGGAAGTGAGC-3′	407	1
	AS	5′-GAAAAGTCCACCCAACTCCATC-3′		
RARβ 2	S	5′-CAGGCTTTTAGCTGGCTTGTCT-3′	436	1
	AS	5′-CCTTTGTCTTGCTACCAATGCA-3′		
RARβ 3	S	5′-CCCATTCTTGCTAGTGTTATTG-3′	260	2
	AS	5′-ACTGAATTCACCACACTTATGG-3′		
RARβ 4	S	5′-GGTTGGCTTTGATTTCTGATGA-3′	321	3
	AS	5′-CCTTGGCAAGATTTCGTTAGTG-3′		
RARβ 5	S	5′-AGCCTTCAGCGACCCCTGATGTG-3′	324	4
	AS	5′-TGCCAGGCCCAGTGCAAAGTGT-3′		
RARβ 6	S	5′-CTCCCCTCCTATAGAGCTTCCCG-3′	315	5
	AS	5′-AS CAATGTCTCTTGGTCCCCTCCC-3′		
RARβ 7	S	5′-CTGGTTATCTGTCATAGCTTAACTCC-3′	359	8
	AS	5′-TCAGTCCAAAAACTAAGCAGCA-3′		

In the table, S indicates the sense strand and AS indicates the antisense strand.

### Single nucleotide polymorphism genotyping by sequencing

After the PCR amplification products were purified using the PCR Cleanup Kit (Axygen Biosciences, Union City, CA), nucleotide sequence analysis was performed with an ABI 3700 sequencer (Applied Biosystems Inc., Foster City, CA). Using Clustal X (version 13.0), we compared all sequencing results to identify variations and to check the sequence map of mutable points to confirm the genotype. Positive results were further confirmed by reverse sequencing. Mutation frequencies of more than 1% were defined as the SNPs.

### Analysis of SNPs

Allelic frequencies of all detected SNPs were assessed for Hardy–Weinberg equilibrium. The frequency (P) of allele A was calculated as PA=(2×NAA+NAB)/(2×N) where NAA was the number of wildtypes, NAB was the number of heterozygotes, and N was the sample size of the group. The frequency of allele B was calculated as PB=1-PA. The theoretical value of three genotypes was calculated according to the allele frequency: NAA(E)=N×(PA^2^),NAB(E)=2N×PA×PB,NBB(E)=N×(PB^2^) where (E) was the expected value and NBB was the number of homozygous mutations. The Hardy–Weinberg equilibrium for the genotype distributions was examined by the χ^2^ test in each group, where χ^2^=[NAA- NAA(E)]^2^/ NAA(E) + [NAB-NAB(E)]^2^/ NAB(E) +[NBB- NBB(E)]^2^/ NBB(E). The level of Hardy–Weinberg equilibrium was set at p>0.05. All samples were assumed to be from the same Mendelian population. Differences in the observed genotype and allelic frequencies between the high myopia and the control subjects were also examined by the χ^2^ test. Statistical analyses were performed with the SPSS software (version 13.0 for Windows; SPSS Science Inc., Chicago, IL). The power analysis for the χ^2^ test was run using SAS software (version 9.0; SAS Institute, Cary, NC).

## Results

### Clinical characteristics

The age range of high myopia onset for most cases (78.7%) was 5–17 years. The ocular characteristics of spherical power, astigmatism and axial length were similar for left and right eyes (Table 1). Of the 175 high myopia cases, almost all exhibited some level of pathological change in the fundus, and some had vision-threatening complications. Leopard-like fundus was the most common sign (45%), followed by conus (22.5%), and chorioretinal atrophy (22.5%). Other changes included complicated cataract (20%), retinal degenerations (8.3%), posterior staphyloma (7.5%), lacquer cracks (6.7%), vitreous opacities (5.8%), retinal hole (5.8%), retinal detachment (4.2%), macular hemorrhage (3.4%), and choroidal neovascularization (0.8%).

### Genetic association study

**Figure 1:**
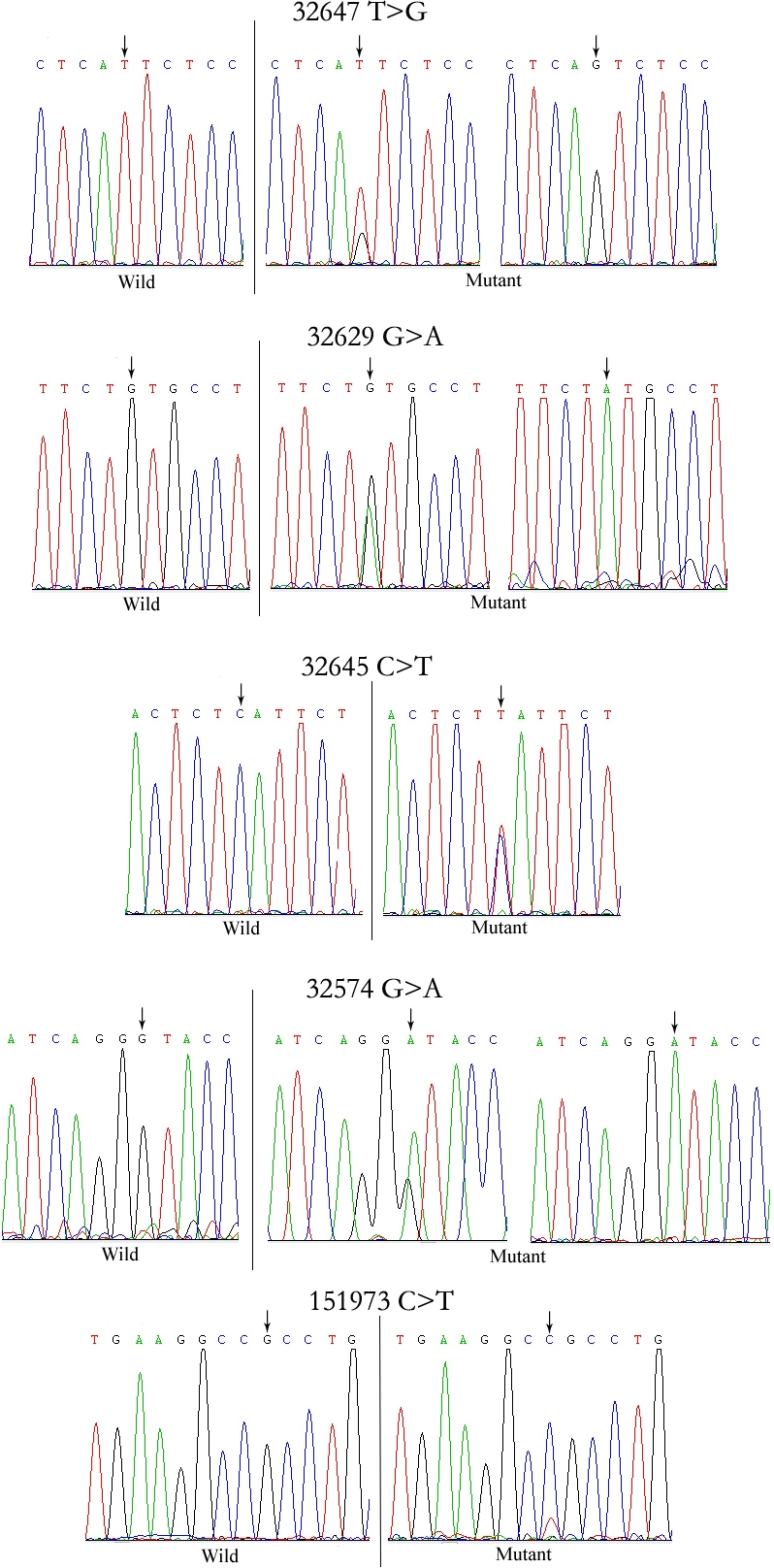
Five SNPs identified in the *RARβ* gene. Variant 32647T>G (NCBI notes as rs58244688 and rs2067964) was a transversion of pyrimidine to purine or purine to pyrimidine. The other four were transitions. Variant 32629G>A, 32645C>T, and 32647T>G were located in introns, and 32574G>A and 151973C>T were located in the coding regions.

**Figure 2:**
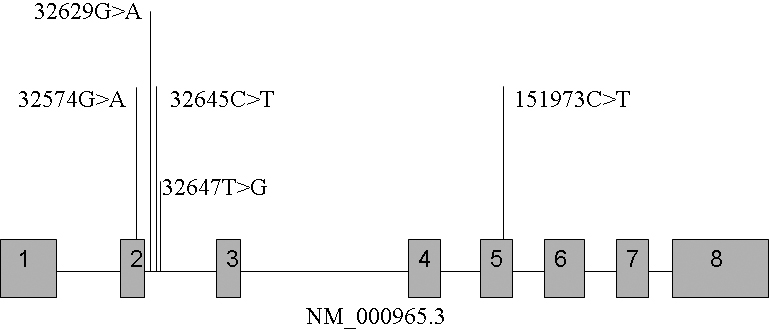
Schematic diagram of the five single nucleotide polymorphisms within the *RARβ* gene. The gray rectangles represent exons and the lines represent introns.

We detected six exons and some flanking regions of the *RARβ* gene. There were five SNPs: 32574G>A, 32629G>A, 32645C>T, 32647T>G, 151973C>T (Table 3, Figure 1). Of these, only one, 32647T>G, had been reported in the National Center for Biotechnology Information (NCBI) dbSNP database. This intron SNP (NCBI notes as rs58244688 and rs2067964) was a transversion, converting pyrimidine to purine or purine to pyrimidine. The other four were transitions with 32629G>A and 32645C>T located in introns and 32574G>A and 151973C>T located in coding regions (Figure 2). Neither of these SNPs affected the amino acid sequence.

**Table 3:** SNPs detected in the *RARβ* gene.

**SNP**	**High myopia group n (%)**	**Control group n (%)**	**χ^2^**	**p value**
32574G>A	6 (3.59%)	5 (5.05%)	0.333	0.564
32629G>A	26 (15.57%)	14 (14.14%)	0.099	0.753
32645C>T	3 (1.80%)	2 (2.02%)	0.017	0.897
32647T>G	29 (17.37%)	20 (20.20%)	0.108	0.743
151973C>T	3 (1.84%)	1 (1.04%)	0.269	0.604

The power analysis for χ^2^ test using SAS indicated the number of participants was adequate.

The variation frequency of the SNPs in the high myopia and the control subjects were compared using the χ^2^ test. There were no significant differences for the genotype and allelic frequencies for the five SNPs between the high myopia and the control subjects (Table 4). Based on the genotype frequencies, the majority of SNP genotypes were heterozygous. All detected SNPs were assessed for Hardy–Weinberg disequilibrium by the χ^2^ test. For all subjects, the distributions of four of the SNP *RARβ* genotypes were within the predictions of the Hardy–Weinberg equilibrium (p>0.05, Table 4). However, one of them, 32574G>A in the high myopia group, was not distributed accordingly (p<0.0005).

**Table 4:** Observed frequency of the SNPs in high myopia and control groups.

** **	**High myopia group**					**Control group**				
**SNPs**	**AA**	**AB**	**BB**	**HWD χ^2^**	**p value**	**AA**	**AB**	**BB**	**HWD χ^2^**	**p value**
32574G>A	161	5	1	12.213	0.0005*	94	5	0	0.066	0.80
32629G>A	141	25	1	0.009	0.92	85	14	0	0.573	0.45
32645C>T	164	3	0	0.014	0.91	97	2	0	0.010	0.92
32647T>G	136	30	1	0.227	0.63	79	19	1	0.015	0.90
151973C>T	160	3	0	0.014	0.91	95	1	0	0.003	0.96

In the table, AA indicates genotype with homozygous normal allele, AB indicates genotype with heterozygous sequence alterations, and BB indicates genotype with homozygous sequence alterations. HWD χ^2^ indicates Hardy–Weinberg equilibrium χ^2^ statistic.

## Discussion

SNPs are the most common type of human genetic variation. They can be identified using various methods, such as direct sequencing, denaturing high performance liquid chromatography, restriction enzyme digestion, and others. Direct genomic DNA sequencing yields high fidelity results with less effort. In this study, six exons and a few introns in *RARβ* were detected by direct sequencing. Five SNPs were found, of which four (32574G>A, 32629G>A, 32645C>T, and 151973C>T) had not been described previously. Most of the *RARβ* SNPs were heterozygous, and there were no differences in frequency between high myopia patients and normal subjects. As a result, there was no association between DNA sequence variation of *RARβ* and high myopia. Two of the SNPs were located in the coding regions of *RARβ*, and the distribution of one of these in the high myopia group, 32574G>A, was different from that predicted by the Hardy–Weinberg equilibrium. While neither SNP affected the amino acid sequence of the protein, these variations could affect mRNA transcription and/or the secondary structure of the mRNA. Such changes could result in altered expression levels of the RARβ protein [31].

By modulating cell proliferation, differentiation, and cell fate, RA is an important regulator of ocular embryonic and post-embryonic development [27,32]. It is a critical molecule in cultured embryonic photoreceptors as it promotes survival of neurons. It also promotes differentiation of photoreceptors but decreases differentiation of amacrine cells [33,34]. The RA content increases in the retina of chicks within 5 days after initiation of deprivation myopia, and ocular elongation is induced by oral RA in chicks and guinea pigs [23-25,35]. Retinoic acid activates *RARβ* [36], and it increases the expression of *RARβ* and downstream genes. *RARβ2*/*RARγ2* double null mutant mice are characterized by retinal dysplasia, abnormal development of the choroid and sclera, and other eye defects [37]. Given the critical role that RA plays in scleral remodeling and the development of myopia, it is likely RA receptors, including RARβ, are important in mediating these changes.

Although RA and RARs are clearly important in the development of myopia in animal models, we did not find any association between the DNA sequence variations of *RARβ* and high myopia in the current study. Similarly, no variations in the nucleotide sequence of *RARα* were associated with myopia or hypermetropia [30], although it was differentially expressed during the development of experimental myopia in guinea pigs and chicks [24,38]. In addition, we did not find any genome-wide linkage study on high myopia with a significant log odds score within the chromosomal region containing *RARβ*. Nevertheless, *RARβ* variants could alter the responses to RA and affect downstream regulatory networks that govern the development of the ocular refractive system. While our study focused on exons, SNPs in introns that we did not detect may play a role in high myopia. In addition, expanding the sample size may help find some SNPs that influence the development of high myopia by acting as critical points in the complex pathway causing refractive errors. High myopia is a complex eye disease, and the mechanisms by which RARs contribute to myopia are worth further investigation.
